# A Single Amino Acid in the M1 Protein Responsible for the Different Pathogenic Potentials of H5N1 Highly Pathogenic Avian Influenza Virus Strains

**DOI:** 10.1371/journal.pone.0137989

**Published:** 2015-09-14

**Authors:** Naganori Nao, Masahiro Kajihara, Rashid Manzoor, Junki Maruyama, Reiko Yoshida, Mieko Muramatsu, Hiroko Miyamoto, Manabu Igarashi, Nao Eguchi, Masahiro Sato, Tatsunari Kondoh, Masatoshi Okamatsu, Yoshihiro Sakoda, Hiroshi Kida, Ayato Takada

**Affiliations:** 1 Research Center for Zoonosis Control, Hokkaido University, Sapporo, Japan; 2 Graduate School of Veterinary Medicine, Hokkaido University, Sapporo, Japan; 3 Global Institution for Collaborative Research and Education, Hokkaido University, Sapporo, Japan; 4 School of Veterinary Medicine, the University of Zambia, Lusaka, Zambia; University of Rochester Medical Center, UNITED STATES

## Abstract

Two highly pathogenic avian influenza virus strains, A/duck/Hokkaido/WZ83/2010 (H5N1) (WZ83) and A/duck/Hokkaido/WZ101/2010 (H5N1) (WZ101), which were isolated from wild ducks in Japan, were found to be genetically similar, with only two amino acid differences in their M1 and PB1 proteins at positions 43 and 317, respectively. We found that both WZ83 and WZ101 caused lethal infection in chickens but WZ101 killed them more rapidly than WZ83. Interestingly, ducks experimentally infected with WZ83 showed no or only mild clinical symptoms, whereas WZ101 was highly lethal. We then generated reassortants between these viruses and found that exchange of the M gene segment completely switched the pathogenic phenotype in both chickens and ducks, indicating that the difference in the pathogenicity for these avian species between WZ83 and WZ101 was determined by only a single amino acid in the M1 protein. It was also found that WZ101 showed higher pathogenicity than WZ83 in mice and that WZ83, whose M gene was replaced with that of WZ101, showed higher pathogenicity than wild-type WZ83, although this reassortant virus was not fully pathogenic compared to wild-type WZ101. These results suggest that the amino acid at position 43 of the M1 protein is one of the factors contributing to the pathogenicity of H5N1 highly pathogenic avian influenza viruses in both avian and mammalian hosts.

## Introduction

Influenza A viruses are pleomorphic, enveloped RNA viruses belonging to the family *Orthomyxoviridae* and have a genome with eight segments of negative-strand RNA. Influenza A viruses are divided into subtypes based on the antigenicity of two envelope glycoproteins, hemagglutinin (HA) and neuraminidase (NA) and known to have a wide host range including many avian and mammalian species. Epidemiological studies have revealed that wild water birds, especially migratory ducks, are the natural hosts of influenza A viruses. All of the known subtypes of influenza A viruses, except H17N10 and H18N11 whose genomes were detected in bats [1, 2], have been found in water birds [3, 4]. Furthermore, influenza A viruses circulating in nature are nonpathogenic in ducks and evolutionarily stable, suggesting that these viruses and their natural hosts have reached a long-established adaptive optimum [5, 6].

In 1997, outbreaks of highly pathogenic avian influenza (HPAI) caused by H5N1 viruses occurred at live bird markets in Hong Kong and human cases of H5N1 HPAI virus (HPAIV) infection were reported [7, 8]. Since then, H5N1 HPAIVs have been circulating in poultry for more than a decade [9], and huge numbers of wild and domestic birds have died or been euthanized. In 2002, a large number of water birds, including ducks, geese, and other birds, died due to H5N1 HPAIV infection in Hong Kong [10]. Furthermore, in 2005, approximately 6,000 migratory water birds were found dead with H5N1 HPAIV infection in Qinghai Lake, China [11]. Since then, many cases of fatal HPAIV infection of wild birds, including ducks, have been reported in several countries.

In addition to infection of avian species, it has been reported that H5N1 HPAIVs are occasionally transmitted to humans and cause severe pneumonia with high case mortality rates [12]. Since HPAIV transmission to humans with a fatal outcome was first reported in 1997, 826 human cases of H5N1 HPAIV infection have been reported (as of 31 March in 2015, http://www.who.int/). Although several genetic and molecular factors contributing to HPAIV pathogenicity in mammalian hosts have been reported, the mechanisms underlying the high pathogenic potential of H5N1 HPAIVs in mammalian hosts are not fully understood [13, 14].

HPAIVs are currently restricted to viruses of H5 and H7 HA subtypes that have multiple basic amino acids at their cleavage sites which are recognized by ubiquitous proteases such as furin [15], rendering these viruses capable of causing systematic infection with fatal outcomes in chickens. This mechanism plausibly explains the high lethality of HPAIVs in chickens but not in mice [16]. Interestingly, HPAIVs are, in general, known to show no or only mild pathogenicity for ducks although some H5N1 HPAIV strains are reported to kill them [10, 17]. Thus, other factors in addition to the presence of multiple basic amino acids at the cleavage site of the HA should contribute to the pathogenicity of HPAIVs for some hosts (e.g., mice and ducks) [18].

In 2010–2011, multiple outbreaks of H5N1 HPAI occurred in various parts of Japan [19]. In the same year, before those outbreaks, we isolated two strains of H5N1 HPAIVs, WZ83 and WZ101, from fecal samples of apparently healthy migratory ducks arriving at the northernmost stopover site of the birds in Japan [20]. WZ83 and WZ101 are almost identical genetically and belong to the same clade, which contains several viruses detected over the whole of Japan and Korea [19]. In this study, we found that WZ101 showed higher pathogenicity than WZ83 for chickens, ducks, and mice, despite the high genetic identity between these strains. To determine the molecular basis for the difference in pathogenicity between WZ83 and WZ101, we generated reassortant viruses using a plasmid-based reverse genetics system, and found that the amino acid at position 43 of the M1 protein, previously not identified as a molecular determinant of virulence of influenza A viruses, was the major contributor to the higher pathogenicity of WZ101 in both avian and mammalian species.

## Materials and Methods

### Viruses and cells

Influenza virus strains WZ83 and WZ101 were isolated from fecal samples of healthy migratory ducks arriving at Lake Ohnuma, Wakkanai, Hokkaido, en route south from their nesting areas in Siberia [20]. After isolation, these viruses were passaged once in 10-day-old embryonated chicken eggs at 35°C and stored at -80°C until use. Chicken and duck embryo fibroblasts (CEF and DEF, respectively) were prepared from 10-day-old chicken embryos and 13-day-old duck embryos, respectively, as previously described [21, 22] with slight modification. Madin-Darby canine kidney (MDCK) cells [23], CEF, and DEF were grown in Dulbecco’s minimal essential medium (DMEM) supplemented with 10% calf serum, 100 U/ml penicillin, and 0.1 mg/ml streptomycin. Human embryonic kidney 293T cells [24] were grown in DMEM supplemented with 10% fetal calf serum and antibiotics as described above. All cells were incubated at 37°C in a 5% CO_2_ atmosphere except otherwise indicated.

### Construction of plasmids

Viral RNAs of WZ83 and WZ101 were extracted from infectious allantoic fluid using a QIAamp Viral RNA Mini Kit (Qiagen) and reverse transcribed with Moloney murine leukemia virus reverse transcriptase (Invitrogen) using the uni12 primer (5’-AGCAAAAGCAGG) [25]. Then, for the expression of vRNAs, cDNAs of WZ83 and WZ101 gene segments were cloned into the pHH21 vector, which contains the human RNA polymerase I promoter and the mouse RNA polymerase I terminator separated by BsmBI sites [26]. For the viral protein expression, the cDNAs encoding the PB2, PB1, PA, and NP genes of WZ83 and WZ101 were cloned into the multiple-cloning site of the eukaryotic expression vector pCAGGS/MCS (controlled by the chicken β-actin promoter) [27]. All constructed plasmids were sequenced to confirm the absence of unexpected mutations.

### Generation of viruses from plasmids

**Fig 1:**
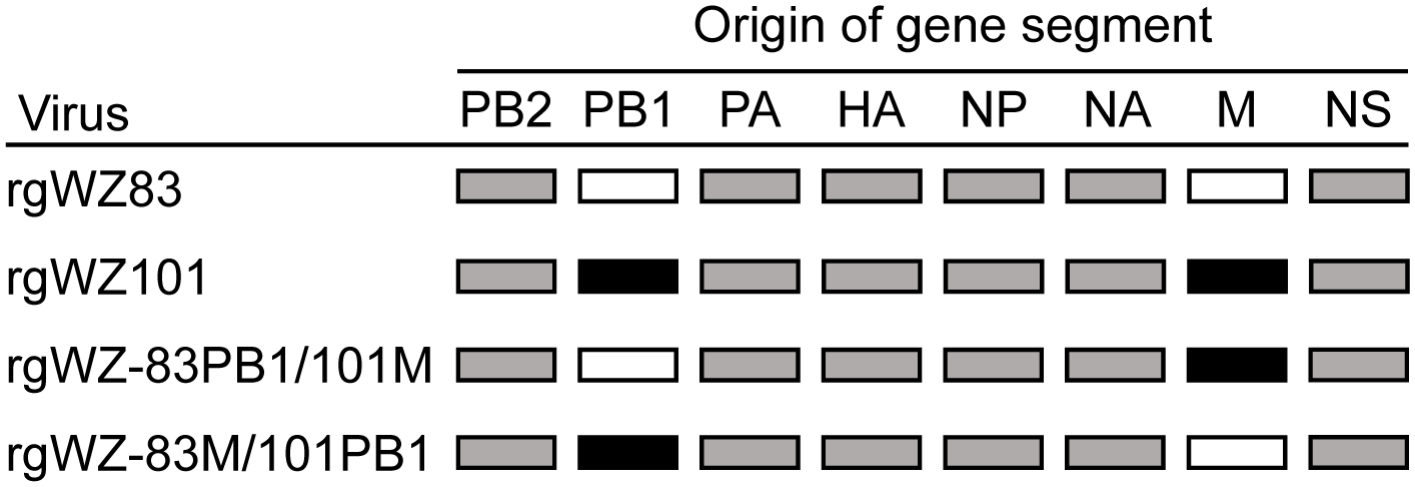
Viruses used in this study. Wild-type (rgWZ83 and rgWZ101) and reassortant viruses (rgWZ-83PB1/101M; WZ83 whose M gene was replaced with that of WZ101 and rgWZ-83M/101PB1; WZ101 whose M gene was replaced with that of WZ83) were generated as described in Materials and Methods.

Wild-type rgWZ83 and rgWZ101 and their reassortant viruses (rgWZ-83PB1/101M; WZ83 whose M gene was replaced with that of WZ101 and rgWZ-83M/101PB1; WZ101 whose M gene was replaced with that of WZ83) (Fig 1) were generated by reverse genetics as described previously [26] with slight modification. Briefly, 293T cells were transfected with 1 μg of each of the 12 plasmids (8 plasmids for viral RNA expression and 4 plasmids for viral polymerase and NP protein expression) encoding WZ83 and WZ101 genes using TransIT-LT1 (Mirus Bio) according to the manufacturer’s protocol. Forty-eight hours after transfection, the supernatant of transfected 293T cells was collected, diluted at 1:10, and transferred to confluent monolayers of MDCK cells. Rescued viruses were propagated in MDCK cells and titers were determined as plaque-forming units (PFU) using MDCK cells and a 50% tissue culture infectious dose (TCID_50_) using CEF, DEF, and MDCK cells. The genomes of the rescued viruses were sequenced to confirm the absence of unexpected mutations.

### Experimental infection of chickens, ducks, and mice

All experimental protocols were reviewed and approved by the Hokkaido University Animal Care and Use Committee. Four-week-old chickens (Boris Brown), 2-week-old domestic ducks (Cherry Valley, kindly provided by Takikawa Shinseien, Hokkaido, Japan), and 7-week-old female mice (BALB/c) were used to assess the pathogenicity of the viruses. Eight chickens in each group were intravenously infected with each virus (10^6^ PFU/bird) and monitored clinically at 8-hour intervals over a period of 6 days. Six chickens in each group were intranasally infected with each virus (10^6^ PFU/bird) and monitored clinically at 24-hour intervals over a period of 14 days. Five ducks in each group were infected intranasally with each virus (10^6^ PFU/bird) and monitored clinically at 12-hour intervals over a period of 14 days. Clinical signs of infected ducks were evaluated and scored daily on a scale of 0–3 (0 = healthy; 1 = mild ill; 2 = severe ill; 3 = dead) during the observation period. Birds exhibiting severe disease signs (i.e. severe neurological symptoms or paralysis) were euthanized with isoflurane and death was recorded as occurring the next day. Three mice in each group were intranasally infected with each virus (10, 10^2^, 10^3^, and 10^4^ PFU/head), and their body weights and survival (fatality) rates were monitored at 24-hour intervals over a period of 14 days. Mice with body weight loss of more than 25% of their original body weight were euthanized with isoflurane and death was recorded as occurring the next day. These animal experiments were carried out in the biosafety level 3 facility at the Hokkaido University Research Center for Zoonosis Control, Japan. Statistical significance of survival time for chickens that died during the experimental period and the mean clinical score of individual ducks were calculated using student’s t-test with Bonferroni correction.

### Growth kinetics of viruses in DEF, CEF, and MDCK cells

Cultured DEF, CEF, and MDCK cells were infected with each virus at a multiplicity of infection (MOI) of 0.001. After adsorption for 1 hour, the inoculum was removed and cells were washed and overlaid with Eagle’s minimal essential medium (MEM) containing bovine serum albumin (0.3%), penicillin (100 U/ml), and streptomycin (0.1 mg/ml). DEF, CEF, and MDCK cells were incubated at 40°C or 35°C, and the culture supernatants were collected at various time points. The titers of viruses released into cell culture supernatants were determined by plaque assays in MDCK cells.

### Cycloheximide treatment and detection of M1 protein in infected CEF

CEF were infected with each virus at an MOI of 2 and incubated for 6 hours. Then the culture medium was replaced by MEM supplemented with bovine serum albumin (0.3%), penicillin (100 U/ml), and streptomycin (0.1 mg/ml) containing cycloheximide (CHX) (100 μg/ml) or ethanol (solvent for CHX) and the incubation was continued for further 6 hours at 37°C. Cells were lysed in sodium dodecyl sulfate (SDS) buffer containing 2-mercaptoethanol (2-ME), and the M1 protein and β-actin in whole cell lysate was detected as described below. Samples were heated for 5 minutes at 98°C and analyzed by 10% SDS-polyacrylamide gel electrophoresis (SDS-PAGE). After electrophoresis, separated proteins were blotted onto a polyvinylidene difluoride membrane (Millipore). Mouse monoclonal antibodies against the M1 protein (APH6-23-1-6) and β-actin (Abcam) were used as primary antibodies to detect the M1 and β-actin, respectively. The bound antibodies were detected with peroxidase-conjugated goat anti-mouse IgG (H+L) (Jackson ImmunoResearch), followed by visualization with Immobilon Western (Millipore). Band intensities of the M1 protein were analyzed with a VersaDocTM Imaging System (Bio-Rad) and Image LabTM software (Bio-Rad).

## Results

### Characterization of WZ83 and WZ101

**Fig 2:**
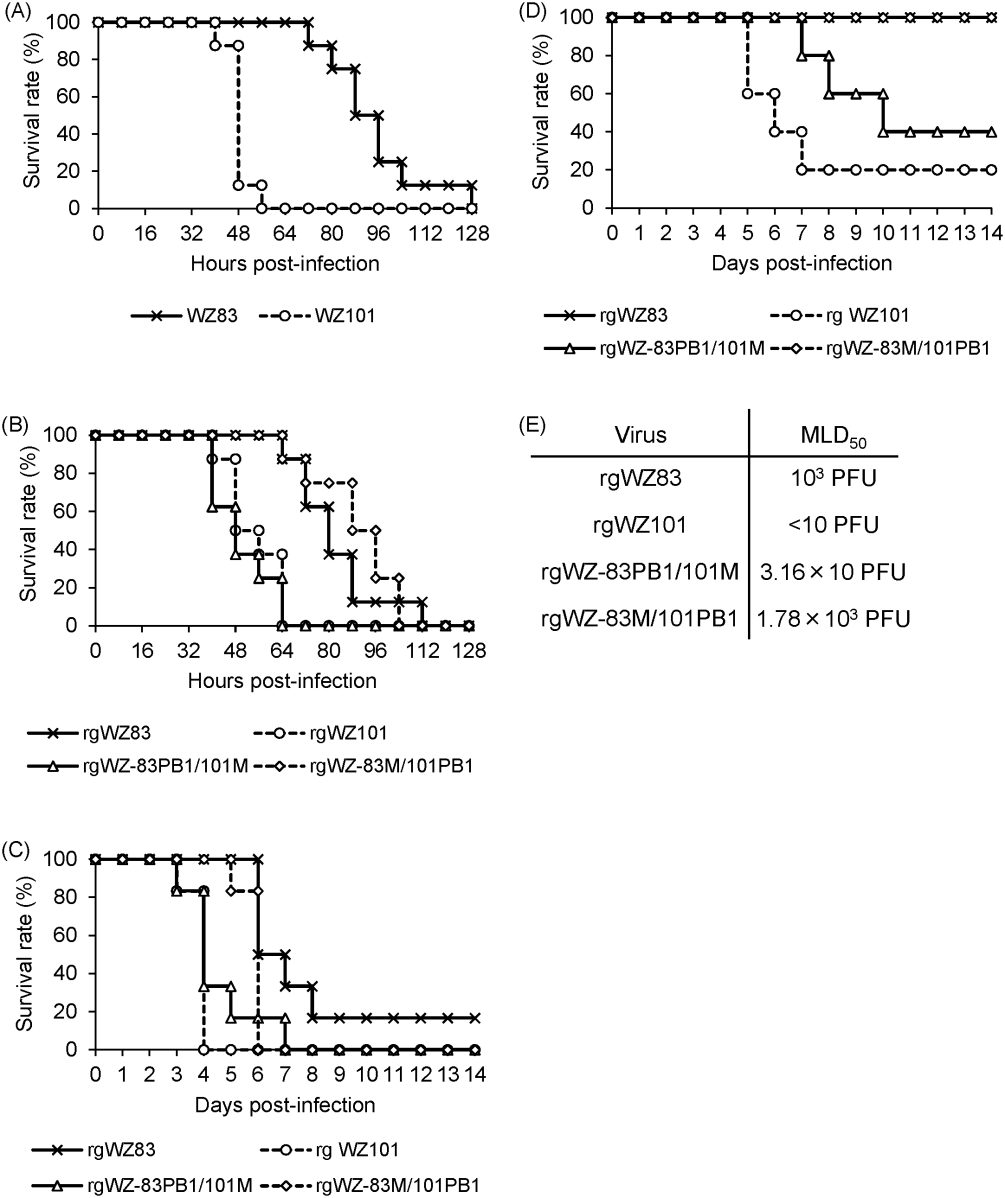
Pathogenicities of H5N1 HPAIVs for chickens, ducks, and mice. Eight chickens of each group were intravenously infected with 10^6^ PFU of 2 original isolates (WZ83 and WZ101) (A). Eight chickens of each group were intravenously infected with 10^6^ PFU of plasmid-derived viruses (rgWZ83, rgWZ101, rgWZ-83PB1/101M, and rgWZ-83M/101PB1) and observed for clinical symptoms every 8 hours (B). Six chickens of each group were intranasally infected with 10^6^ PFU of each virus and observed for clinical symptoms every 24 hours (C). Five ducks of each group were intranasally infected with 10^6^ PFU of rgWZ83, rgWZ101, rgWZ-83PB1/101M, or rgWZ-83M/101PB1 and observed for clinical symptoms every 12 hours (D). Three mice of each group were intranasally infected with one of 4 infectious doses (10, 10^2^, 10^3^, or 10^4^ PFU) of each virus, and MLD_50_ was calculated (E).

We first found that chickens infected intravenously with WZ83 or WZ101 uniformly showed loss of appetite, lethargy, subcutaneous hemorrhages in the legs, edema of the face and legs, and paralysis. However, it was noted that WZ101 killed the chickens more rapidly than WZ83; chickens infected with WZ83 died at 72–128 hours post-infection (hpi) whereas those infected with WZ101 died at 40–56 hpi (Fig 2A) (p<0.001 for the comparison of the mean death time). We then sequenced all the RNA segments and found that WZ83 and WZ101 were almost identical and differed by only two amino acids in M1 and PB1 proteins, the primary products of the M and PB1 RNA segments. WZ83 and WZ101 have isoleucine (Ile) and methionine (Met) at amino acid position 43 of M1, and Ile and Met at amino acid position 317 of PB1, respectively. We found no difference in the M2 and PB1-F2 proteins, which were also expressed by the respective gene segments.

### Pathogenicities of rgWZ83, rgWZ101, rgWZ-83PB1/101M, and rgWZ-83M/101PB1 in chickens

To investigate the genetic basis of the difference in pathogenicity between WZ83 and WZ101, we established a reverse genetics system for these viruses and rescued wild-type and their reassortant viruses (Fig 1). Intravenous inoculation of these viruses caused similar clinical symptoms and 100% mortality in 4-week-old chickens. Consistent with the difference between the original strains (Fig 2A), rgWZ101 killed chickens more rapidly than rgWZ83 (i.e., while chickens infected with rgWZ83 died at 64–112 hours post-infection (hpi), those infected with rgWZ101 died at 40–64 hpi) (p<0.01 for the comparison of the mean death time between rgWZ83 and rgWZ101). Interestingly, the pathogenicities of rgWZ-83PB1/101M and rgWZ-83M/101PB1 were similar to those of rgWZ101 and rgWZ83, respectively (i.e., chickens infected with rgWZ-83PB1/101M died at 40–64 hpi whereas those infected with rgWZ-83M/101PB1 died at 64–104 hpi) (Fig 2B) (p<0.01 for the comparison of the mean death time between rgWZ83 and rgWZ-83PB1/101M and between rgWZ101 and rgWZ-83M/101PB1). The pathogenicities of these viruses were also investigated in chickens infected intranasally (Fig 2C). We found that all 4 viruses were highly lethal in intranasally infected chickens, and there was an appreciable difference between rgWZ83 and rgWZ101, as was the case with intravenously infected chickens (p<0.001 for the comparison of the mean death time between rgWZ83 and rgWZ101). Chickens infected with rgWZ101 or rgWZ-83PB1/101M died at 3–7 days post-infection (dpi), whereas chickens infected with rgWZ83 or rgWZ-83M/101PB1 died at 5–8 dpi except one rgWZ83-infected chicken surviving during the 14-day observation period (Fig 2C) (p<0.01 for the comparison of the mean death time between rgWZ83 and rgWZ-83PB1/101M and between rgWZ101 and rgWZ-83M/101PB1). These results indicated that the M gene of WZ101 contributed to the higher pathogenicity of rgWZ101 in chickens.

### Pathogenicities of rgWZ83, rgWZ101, rgWZ-83PB1/101M, and rgWZ-83M/101PB1 in ducks

We then compared the pathogenicities of all 4 rescued viruses in ducks (Fig 2D). All ducks infected with rgWZ83 survived and showed no or only mild clinical symptoms (e.g. somnolentia and lethargy) during the 14-day observation period. In contrast, ducks infected with rgWZ101 showed loss of appetite, lethargy, torticollis, uncontrollable shaking, marked loss of balance, and paralysis, and 4 of the 5 ducks died at 5–7 dpi (p<0.01 for the comparison of the mean clinical score between rgWZ83 and rgWZ101). Unlike rgWZ83, rgWZ-83PB1/101M caused severe clinical symptoms in all ducks and killed 3 of the 5 infected ducks at 7–10 dpi (p<0.01 for the comparison of the mean clinical score between rgWZ83 and rgWZ-83PB1/101M). Interestingly, rgWZ-83M/101PB1 did not kill any ducks and caused no or only mild clinical symptoms, as was the case with rgWZ83 (p<0.01 for the comparison of the mean clinical score between rgWZ101 and rgWZ-83M/101PB1). These results indicated that the single amino acid difference in the M1 protein was a critical factor associated with the lethality of WZ101 in ducks.

### Pathogenicities of rgWZ83, rgWZ101, rgWZ-83PB1/101M, and rgWZ-83M/101PB1 in mice

We further compared the pathogenicities of these viruses in mice (Fig 2E). Four infectious doses (10, 10^2^, 10^3^, or 10^4^ PFU) of each virus were inoculated intranasally into mice (three mice for each dose). We found that rgWZ101 killed all the mice, even at the lowest dose tested (10 PFU), whereas a much higher dose (10^4^ PFU) was required for rgWZ83 to kill all three mice. Both rgWZ-83PB1/101M and rgWZ-83M/101PB1 were lethal for mice at the highest dose (10^4^ PFU) but not at the lowest dose (10 PFU). Interestingly, at the moderate dose (10^2^ PFU), rgWZ-83PB1/101M was lethal and killed all three mice, whereas rgWZ-83M/101PB1 killed no mice. The 50% mouse lethal doses (MLD_50_) of rgWZ83, rgWZ101, rgWZ-83PB1/101M, and rgWZ-83M/101PB1 were 10^3^, <10, 3.16×10, and 1.78×10^3^ PFU, respectively. These results suggested that the single amino acid difference in the PB1 protein, in addition to the M1 protein, was also involved in the higher pathogenicity of WZ101 in mice.

### Growth kinetics of rgWZ83, rgWZ101, rgWZ-83M/101PB1, and rgWZ-83PB1/101M in CEF, DEF, and MDCK cells

**Fig 3:**
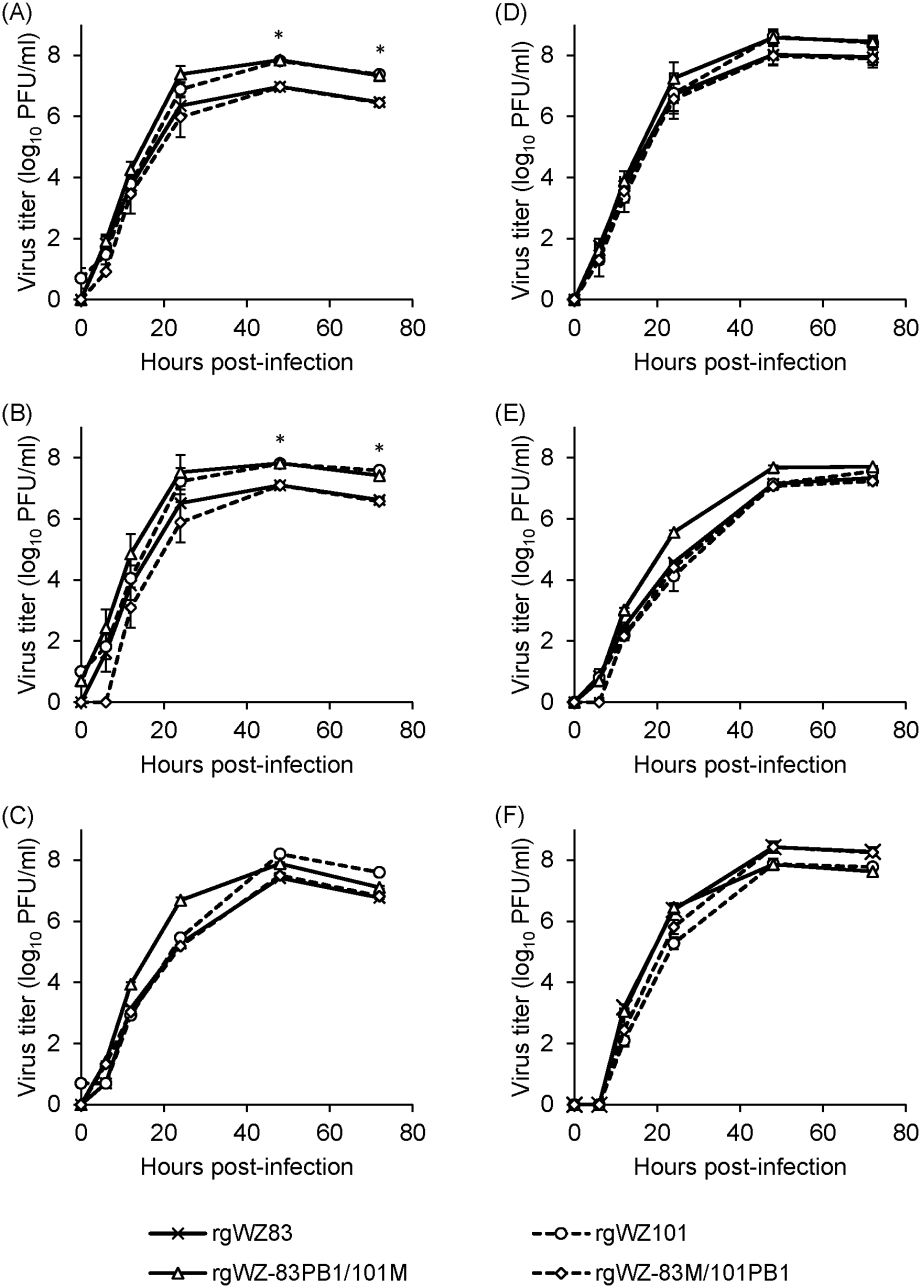
Comparison of growth kinetics of H5N1 HPAIVs in cultured cells. Growth kinetics of rgWZ83, rgWZ101, rgWZ-83PB1/101M, and rgWZ-83M/101PB1 in CEF (A and D), DEF (B and E), and MDCK cells (C and F) at 40°C (A, B, and C) and 35°C (D, E, and F) were compared. The results are presented as the averages and standard deviations of three independent experiments. Statistical significance was calculated using student’s t-test for the comparison between rgWZ83 and rgWZ101, rgWZ83 and rgWZ-83PB1/101M, rgWZ101 and rgWZ-83M/101PB1, and rgWZ-83PB1/101M and rgWZ-83M/101PB1 with Bonferroni correction for time points 48 and 72 hpi (*p<0.01 for all comparisons).

To compare the in vitro replication capacities of the viruses, we tested their growth kinetics in CEF, DEF, and MDCK cells at 40°C and 35°C. We found that rgWZ101 replicated more efficiently than rgWZ83 in CEF and DEF at 40°C, as indicated by approximately 10-fold higher titers than those of rgWZ83 at 48 and 72 hpi (Fig 3A and 3B). The growth kinetics of rgWZ-83PB1/101M and rgWZ-83M/101PB1 were similar to those of rgWZ101 and rgWZ83 in CEF and DEF, respectively (Fig 3A and 3B). Interestingly, the difference was less significant when the viruses were grown in CEF and DEF at 35°C (Fig 3D and 3E). In MDCK cells, rgWZ101 and rgWZ-83PB1/101M showed higher titers than rgWZ83 and rgWZ-83M/101PB1 at several time points when grown at 40°C (Fig 3C). However, unlike the growth kinetics in CEF and DEF, overall differences between the viruses were limited and not clear in MDCK cells.

### Difference in the stability of the M1 proteins in cultured cells

**Fig 4:**
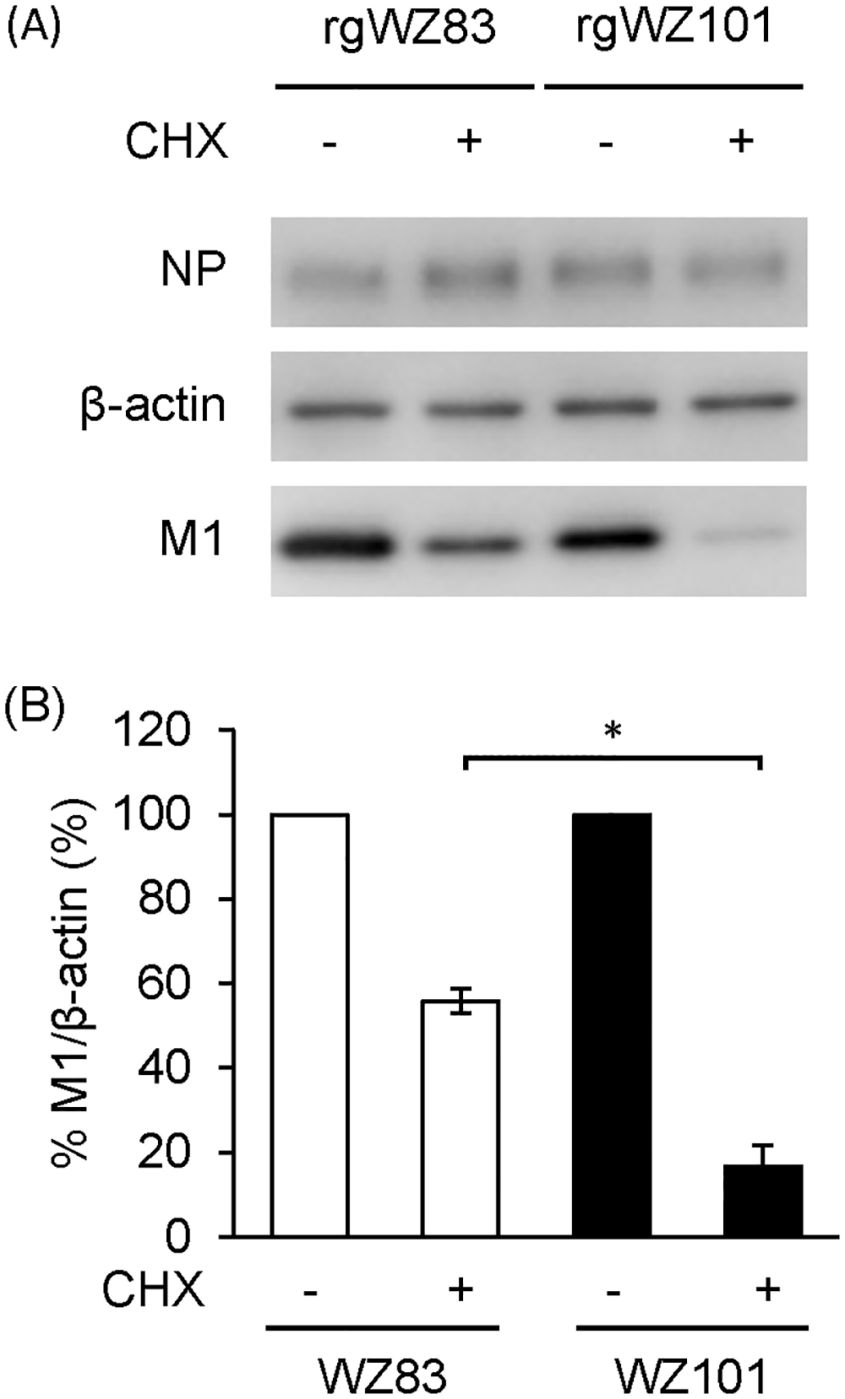
Stability of the M1 protein in cultured cells. CEF were infected with rgWZ83 and rgWZ101 and incubated for 6 hours. Then cells were treated with CXH and further incubated for 6 hours. The amount of the M1 protein was measured by western blotting and β-actin was used as a loading control. Representative data of three independent experiments are shown (A). Relative percentages of band intensities of the M1 protein are presented as the averages and standard deviations of three independent experiments (B). Statistical significance was calculated using student’s t-test (*p<0.01).

Since the M1 protein was shown to be important for the difference in the pathogenicity and replication capacity between WZ83 and WZ101, we then sought to find the different biological properties of the M1 protein among the strains. We focused on the stability of the M1 protein since we hypothesized that it might be involved in viral pathogenesis. rgWZ83- and rgWZ101-infected CEF were incubated for 6 hours followed by incubation with CHX for 6 more hours, and the amounts of the M1 protein were compared (Fig 4A and 4B). Without CHX treatment, similar amounts of the M1 protein were detected in CEF infected with rgWZ83 and rgWZ101. As expected, reduced amounts of the M1 protein of rgWZ83 and rgWZ101 were detected in infected CEF incubated with CHX. Interestingly, we found that the amount of the rgWZ101 M1 protein was significantly lower than that of the rgWZ83 M1 protein in CHX-treated CEF. These results suggested that the WZ101 M1 protein was more unstable and degraded more rapidly in CEF than the WZ83 M1 protein.

## Discussion

In this study, we found that two H5N1 HPAIV strains isolated in Japan, WZ83 and WZ101, were genetically almost identical but had different pathogenicities. We demonstrated that rgWZ101 had higher pathogenicity than rgWZ83 in chickens, ducks, and mice and that the amino acid at position 43 in the M1 protein, not reported previously to be associated with virulence of influenza A viruses, was responsible for the higher pathogenicity of WZ101. We further demonstrated that this amino acid affected the in vitro replication capacity of WZ83 and WZ101 and the stability of the M1 protein in infected cells.

Based on sequences obtained from the NCBI Influenza Virus Resource Database, the amino acid at position 43 of the M1 protein is highly conserved among influenza A viruses (i.e., 1194 of 1201 viruses isolated from avian species and 2648 of 2662 viruses isolated from all hosts have Met at this position of the M1 protein). In 2010 and 2011, many strains of the H5N1 HPAIV closely related to WZ83 and WZ101 were isolated not only in Japan but also in China, Mongolia, Russia, and Korea. However, all of these viruses except WZ83 have Met at amino acid position 43 of the M1 protein. These observations suggest that Met at amino acid position 43 of the M1 protein is important for the viral life cycle and/or not exposed to selection pressure. Importantly, however, WZ83 still retained high pathogenicity in chickens and high replication capacity in vitro, indicating that the substitution at this position might not affect the fundamental functions of the M1 protein.

The M1 protein is a multifunctional protein playing many essential roles throughout the viral replication cycle. It forms the major structural component of influenza virus particles, plays an essential role in viral assembly associated with influenza virus ribonucleoprotein (RNP) and RNA [28–32], functions in transcription inhibition [33], and controls RNP nuclear import and export [34–38]. However, little has been reported about the direct contribution of the M1 protein to the pathogenicity of H5N1 HPAIVs. Two amino acid residues at positions 30 and 215 of the M1 protein were reported to be important for the pathogenicity of H5N1 HPAIVs in mice [13]. While these 2 amino acids are located in the second helix and C-terminal domain of the M1 protein, respectively, amino acid position 43, which is responsible for the difference in the pathogenicity between WZ83 and WZ101, is located in the N-terminal domain and fold into the third helix of the M1 protein [39, 40]. The amino acid substitution at position 41 of the M1 protein was reported to be associated with increased replication and virulence of human influenza viruses in mice, suggesting a role in host adaptation [41]. This amino acid residue was also implicated in determining the virion morphology (i.e., filamentous or spherical) of influenza viruses [42, 43], while both rgWZ83 and rgWZ101 virions were spherical (data not shown). These observations suggested the different contributions of these amino acids to the pathogenicities of H5N1 HPAIVs in different animals.

It was also noted that the difference between WZ83 and WZ101 in growth kinetics experiments was most apparent in avian cells at the higher temperature. In general, avian influenza viruses replicate at higher temperatures than human influenza viruses, most likely due to higher temperature of avian species compared to mammals. It was shown that in vitro replication capacity at higher temperature (41°C) was correlated with the pathogenicity of some HAPIV strains in chickens [44]. Therefore, efficient replication of WZ101 at higher temperature is likely linked to its increased pathogenicity particularly in chickens and ducks. It was also suggested that the M gene segment of a live attenuated human influenza vaccine strain affected temperature sensitivity of the virus [45]. Although detailed mechanisms are not clear, we assume that amino acid position 43 is involved in the WZ101 thermal stability which might contribute to the pathogenicity.

It has been suggested that difference in viral protein degradation in infected cells could affect the pathogenicity of the rabies virus [46]. A rabies virus strain with a higher degradation rate of its G protein induced less apoptosis in infected neuronal cells and had a 50% higher pathogenicity index for mice than a variant strain with a lower degradation rate of the G protein [46]. It is known that the M1 protein of influenza A viruses mediates activation of caspase, which plays essential roles in inducing apoptosis both in viral infected and M1-expressing cells [47]. Although the transient expression of WZ83 and WZ101 M1 proteins in 293T and Hela cells did not affect the caspase activity (data not shown), our data suggested that the WZ101 M1 protein was more unstable and degraded more rapidly in virus-infected CEF than the WZ83 M1 protein. Thus, it might still be possible that rapid degradation of the WZ101 M1 protein results in weaker anti-viral host responses due to reduced apoptosis and/or antigen presentation of infected cells.

In summary, we demonstrated that the amino acid at position 43 of the M1 protein could be a critical factor contributing to high pathogenicities of H5N1 HPAIVs for both avian and mammalian species, although the underlying mechanisms remain to be determined. It is noteworthy that the amino acid difference at this position particularly affected viral pathogenicity in ducks. Our data underscore the need for continued global monitoring of H5N1 HPAIVs for early detection of HPAIVs with reduced virulence to ducks due to the substitution at this amino acid position, enabling us to take preemptive measures to minimize the risk of their transmission to domestic poultry. It is also important to investigate whether such viruses are naturally selected and become predominant in wild duck populations.

## References

[1] Tong S, Li Y, Rivailler P, Conrardy C, Castillo DA, Chen LM, et al A distinct lineage of influenza A virus from bats. Proc Natl Acad Sci U S A. 2012;109(11):4269–74. 10.1073/pnas.1116200109 22371588 PMC3306675

[2] Tong S, Zhu X, Li Y, Shi M, Zhang J, Bourgeois M, et al New world bats harbor diverse influenza A viruses. PLoS Pathog. 2013;9(10):e1003657 10.1371/journal.ppat.1003657 24130481 PMC3794996

[3] Ito T, Okazaki K, Kawaoka Y, Takada A, Webster RG, Kida H. Perpetuation of influenza A viruses in Alaskan waterfowl reservoirs. Arch Virol. 1995;140(7):1163–72. 7646350 10.1007/BF01322743

[4] Okazaki K, Takada A, Ito T, Imai M, Takakuwa H, Hatta M, et al Precursor genes of future pandemic influenza viruses are perpetuated in ducks nesting in Siberia. Arch Virol. 2000;145(5):885–93. 10881676 10.1007/s007050050681

[5] Kida H, Kawaoka Y, Naeve CW, Webster RG. Antigenic and genetic conservation of H3 influenza virus in wild ducks. Virology. 1987;159(1):109–19. 2440178 10.1016/0042-6822(87)90353-9

[6] Kida H, Yanagawa R, Matsuoka Y. Duck influenza lacking evidence of disease signs and immune response. Infect Immun. 1980;30(2):547–53. 7439994 10.1128/iai.30.2.547-553.1980 PMC551346

[7] Shortridge KF, Zhou NN, Guan Y, Gao P, Ito T, Kawaoka Y, et al Characterization of avian H5N1 influenza viruses from poultry in Hong Kong. Virology. 1998;252(2):331–42. 9878612 10.1006/viro.1998.9488

[8] Control CfD, Prevention. Isolation of avian influenza A (H5N1) viruses from humans—Hong Kong, May-December 1997. MMWR Morb Mortal Wkly Rep. 1997;46(50):1204 9414153

[9] Webster RG, Peiris M, Chen H, Guan Y. H5N1 outbreaks and enzootic influenza. Emerg Infect Dis. 2006;12(1):3–8. 16494709 10.3201/eid1201.051024 PMC3291402

[10] Sturm-Ramirez KM, Ellis T, Bousfield B, Bissett L, Dyrting K, Rehg JE, et al Reemerging H5N1 influenza viruses in Hong Kong in 2002 are highly pathogenic to ducks. J Virol. 2004;78(9):4892–901. 15078970 10.1128/JVI.78.9.4892-4901.2004 PMC387679

[11] Chen H, Smith GJ, Zhang SY, Qin K, Wang J, Li KS, et al Avian flu: H5N1 virus outbreak in migratory waterfowl. Nature. 2005;436(7048):191–2. 16007072 10.1038/nature03974

[12] Kerkhove MD. Brief literature review for the WHO global influenza research agenda–highly pathogenic avian influenza H5N1 risk in humans. Influenza Other Respir Viruses. 2013;7(s2):26–33. 24034480 10.1111/irv.12077 PMC9680821

[13] Fan S, Deng G, Song J, Tian G, Suo Y, Jiang Y, et al Two amino acid residues in the matrix protein M1 contribute to the virulence difference of H5N1 avian influenza viruses in mice. Virology. 2009;384(1):28–32. 10.1016/j.virol.2008.11.044 19117585

[14] Fan S, Hatta M, Kim JH, Halfmann P, Imai M, Macken CA, et al Novel residues in avian influenza virus PB2 protein affect virulence in mammalian hosts. Nat Commun. 2014;5:5021 10.1038/ncomms6021 25289523 PMC5841464

[15] Senne DA, Panigrahy B, Kawaoka Y, Pearson JE, Suss J, Lipkind M, et al Survey of the hemagglutinin (HA) cleavage site sequence of H5 and H7 avian influenza viruses: amino acid sequence at the HA cleavage site as a marker of pathogenicity potential. Avian Dis. 1996;40(2):425–37. 8790895

[16] Hatta M, Neumann G, Kawaoka Y. Reverse genetics approach towards understanding pathogenesis of H5N1 Hong Kong influenza A virus infection. Philos Trans R Soc Lond B Biol Sci. 2001;356(1416):1841–3. 11779382 10.1098/rstb.2001.1000 PMC1088559

[17] Kishida N, Sakoda Y, Isoda N, Matsuda K, Eto M, Sunaga Y, et al Pathogenicity of H5 influenza viruses for ducks. Arch Virol. 2005;150(7):1383–92. 15747052 10.1007/s00705-004-0473-x

[18] Song J, Feng H, Xu J, Zhao D, Shi J, Li Y, et al The PA protein directly contributes to the virulence of H5N1 avian influenza viruses in domestic ducks. J Virol. 2011;85(5):2180–8. 10.1128/JVI.01975-10 21177821 PMC3067757

[19] Sakoda Y, Sugar S, Batchluun D, Erdene-Ochir TO, Okamatsu M, Isoda N, et al Characterization of H5N1 highly pathogenic avian influenza virus strains isolated from migratory waterfowl in Mongolia on the way back from the southern Asia to their northern territory. Virology. 2010;406(1):88–94. 10.1016/j.virol.2010.07.007 20673942

[20] Kajihara M, Matsuno K, Simulundu E, Muramatsu M, Noyori O, Manzoor R, et al An H5N1 highly pathogenic avian influenza virus that invaded Japan through waterfowl migration. Jpn J Vet Res. 2011;59(2–3):89–100. 21977732

[21] Zimmermann T, Schaefer W. Effect of p-fluorophenyl-alanine of fowl plague virus multiplication. Virology. 1960;11:676–98. 13847553 10.1016/0042-6822(60)90114-8

[22] Adams S, Xing Z, Li J, Mendoza K, Perez D, Reed K, et al The effect of avian influenza virus NS1 allele on virus replication and innate gene expression in avian cells. Mol Immunol. 2013;56(4):358–68. 10.1016/j.molimm.2013.05.236 23911391

[23] Green IJ. Serial propagation of influenza B (Lee) virus in a transmissible line of canine kidney cells. Science. 1962;138(3536):42–3. 13901412 10.1126/science.138.3536.42

[24] DuBridge RB, Tang P, Hsia HC, Leong PM, Miller JH, Calos MP. Analysis of mutation in human cells by using an Epstein-Barr virus shuttle system. Mol Cell Biol. 1987;7(1):379–87. 3031469 10.1128/mcb.7.1.379-387.1987 PMC365079

[25] Hoffmann E, Stech J, Guan Y, Webster RG, Perez DR. Universal primer set for the full-length amplification of all influenza A viruses. Arch Virol. 2001;146(12):2275–89. 11811679 10.1007/s007050170002

[26] Neumann G, Watanabe T, Ito H, Watanabe S, Goto H, Gao P, et al Generation of influenza A viruses entirely from cloned cDNAs. Proc Natl Acad Sci U S A. 1999;96(16):9345–50. 10430945 10.1073/pnas.96.16.9345 PMC17785

[27] Niwa H, Yamamura K, Miyazaki J. Efficient selection for high-expression transfectants with a novel eukaryotic vector. Gene. 1991;108(2):193–9. 1660837 10.1016/0378-1119(91)90434-d

[28] Baudin F, Bach C, Cusack S, Ruigrok RW. Structure of influenza virus RNP. I. Influenza virus nucleoprotein melts secondary structure in panhandle RNA and exposes the bases to the solvent. EMBO J. 1994;13(13):3158–65. 8039508 10.1002/j.1460-2075.1994.tb06614.x PMC395207

[29] Elster C, Larsen K, Gagnon J, Ruigrok RWH, Baudin F. Influenza virus M1 protein binds to RNA through its nuclear localization signal. J Gen Virol. 1997;78(7):1589–96. 9225034 10.1099/0022-1317-78-7-1589

[30] Watanabe K, Handa H, Mizumoto K, Nagata K. Mechanism for inhibition of influenza virus RNA polymerase activity by matrix protein. J Virol. 1996;70(1):241–7. 8523532 10.1128/jvi.70.1.241-247.1996 PMC189810

[31] Ye ZP, Baylor NW, Wagner RR. Transcription-inhibition and RNA-binding domains of influenza A virus matrix protein mapped with anti-idiotypic antibodies and synthetic peptides. J Virol. 1989;63(9):3586–94. 2474671 10.1128/jvi.63.9.3586-3594.1989 PMC250948

[32] Ye Z, Liu T, Offringa DP, McInnis J, Levandowski RA. Association of influenza virus matrix protein with ribonucleoproteins. J Virol. 1999;73(9):7467–73. 10438836 10.1128/jvi.73.9.7467-7473.1999 PMC104273

[33] Wakefield L, Brownlee GG. RNA-binding properties of influenza A virus matrix protein M1. Nucleic Acids Res. 1989;17(21):8569–80. 2479906 10.1093/nar/17.21.8569 PMC335028

[34] Bui M, Whittaker G, Helenius A. Effect of M1 protein and low pH on nuclear transport of influenza virus ribonucleoproteins. J Virol. 1996;70(12):8391–401. 8970960 10.1128/jvi.70.12.8391-8401.1996 PMC190928

[35] Huang X, Liu T, Muller J, Levandowski RA, Ye Z. Effect of influenza virus matrix protein and viral RNA on ribonucleoprotein formation and nuclear export. Virology. 2001;287(2):405–16. 11531417 10.1006/viro.2001.1067

[36] Martin K, Helenius A. Nuclear transport of influenza virus ribonucleoproteins: the viral matrix protein (M1) promotes export and inhibits import. Cell. 1991;67(1):117–30. 1913813 10.1016/0092-8674(91)90576-k

[37] Whittaker G, Bui M, Helenius A. Nuclear trafficking of influenza virus ribonuleoproteins in heterokaryons. J Virol. 1996;70(5):2743–56. 8627748 10.1128/jvi.70.5.2743-2756.1996 PMC190131

[38] Whittaker G, Kemler I, Helenius A. Hyperphosphorylation of mutant influenza virus matrix protein, M1, causes its retention in the nucleus. J Virol. 1995;69(1):439–45. 7983740 10.1128/jvi.69.1.439-445.1995 PMC188592

[39] Wang S, Zhao Z, Bi Y, Sun L, Liu X, Liu W. Tyrosine 132 phosphorylation of influenza A virus M1 protein is crucial for virus replication by controlling the nuclear import of M1. J Virol. 2013;87(11):6182–91. 10.1128/JVI.03024-12 23536660 PMC3648105

[40] Shishkov AV, Goldanskii VI, Baratova LA, Fedorova NV, Ksenofontov AL, Zhirnov OP, et al The in situ spatial arrangement of the influenza A virus matrix protein M1 assessed by tritium bombardment. Proc Natl Acad Sci U S A. 1999;96(14):7827–30. 10393906 10.1073/pnas.96.14.7827 PMC22146

[41] Ward AC. Virulence of influenza A virus for mouse lung. Virus Genes. 1997;14(3):187–94. 9311563 10.1023/a:1007979709403

[42] Elleman CJ, Barclay WS. The M1 matrix protein controls the filamentous phenotype of influenza A virus. Virology. 2004;321(1):144–53. 15033573 10.1016/j.virol.2003.12.009

[43] Campbell PJ, Kyriakis CS, Marshall N, Suppiah S, Seladi-Schulman J, Danzy S, et al Residue 41 of the Eurasian avian-like swine influenza a virus matrix protein modulates virion filament length and efficiency of contact transmission. J Virol. 2014;88(13):7569–77. 10.1128/JVI.00119-14 24760887 PMC4054412

[44] Rott R, Orlich M, Scholtissek C. Differences in the multiplication at elevated temperature of influenza virus recombinants pathogenic and nonpathogenic for chicken. Virology. 1982;120(1):215–24. 7101726 10.1016/0042-6822(82)90019-8

[45] O'Donnell CD, Vogel L, Matsuoka Y, Jin H, Subbarao K. The matrix gene segment destabilizes the acid and thermal stability of the hemagglutinin of pandemic live attenuated influenza virus vaccines. J Virol. 2014;88(21):12374–84. 10.1128/JVI.01107-14 25122789 PMC4248896

[46] Morimoto K, Hooper DC, Spitsin S, Koprowski H, Dietzschold B. Pathogenicity of different rabies virus variants inversely correlates with apoptosis and rabies virus glycoprotein expression in infected primary neuron cultures. J Virol. 1999;73(1):510–8. 9847357 10.1128/jvi.73.1.510-518.1999 PMC103858

[47] Halder UC, Bhowmick R, Roy Mukherjee T, Nayak MK, Chawla-Sarkar M. Phosphorylation drives an apoptotic protein to activate antiapoptotic genes: paradigm of influenza A matrix 1 protein function. J Biol Chem. 2013;288(20):14554–68. 10.1074/jbc.M112.447086 23548901 PMC3656309

